# Impact of individualized nursing interventions on ventilator weaning and respiratory outcomes in ICU patients with severe pneumonia: A retrospective cohort study

**DOI:** 10.1097/MD.0000000000043355

**Published:** 2025-09-05

**Authors:** Ni Li, Xiaofeng Zhong, Chunlan Zheng, Li Zhao, Sha Hu, Wen Li, Bei Pan, Liangyou Zheng

**Affiliations:** a Department of Tuberculosis Ward 3, Wuhan Pulmonary Hospital, Wuhan, Hubei, China; b Department of Critical Care Medicine, Wuhan No.1 Hospital, Wuhan, Hubei, China; c Department of Nursing, Wuhan Pulmonary Hospital, Wuhan, Hubei, China; d Department of Structural Heart Disease Center, Wuhan University Central South Hospital, Wuhan, Hubei, China.

**Keywords:** mechanical ventilation, pulmonary function, severe pneumonia, targeted nursing, weaning success rate

## Abstract

Severe pneumonia frequently necessitates intensive care, with mechanical ventilation serving as a cornerstone of treatment. Despite its life-saving benefits, ventilator weaning poses considerable challenges and is often associated with complications. This study evaluates the efficacy of a tailored nursing approach in enhancing weaning outcomes and accelerating pulmonary recovery. A retrospective analysis was conducted on 110 intensive care units (ICU) patients with severe pneumonia requiring mechanical ventilation at our institution between January 2022 and July 2024. Participants were stratified into 2 cohorts: an intervention group (n = 55) receiving individualized nursing care – encompassing rigorous disease surveillance, optimized airway management, and structured respiratory rehabilitation – and a control group (n = 55) managed with standard protocols. Key findings demonstrated superior clinical metrics in the intervention group, including faster resolution of fever (*P* < .05), reduced intensive care units (ICU) and hospital stays (*P* < .05), and shorter mechanical ventilation duration (*P* < .05). Notably, the intervention group achieved a 96.36% weaning success rate, significantly outperforming the control group’s 81.82% (*P* = .014). Adverse events were markedly lower in the intervention cohort (10.91% vs 40.00%, *P* = .003), alongside significantly higher patient satisfaction scores (*P* = .026). These results underscore that personalized nursing strategies substantially improve ventilator weaning success, expedite respiratory function restoration, mitigate complications, and elevate care satisfaction. The model presents valuable implications for critical care practice.

## 1. Introduction

Severe pneumonia is one of the leading causes of acute respiratory failure and mortality worldwide, particularly in intensive care units (ICU), where mechanical ventilation is often a critical therapeutic intervention.^[1–3]^ Although mechanical ventilation effectively supports respiratory function, its prolonged use increases the risk of ventilator-associated complications, such as ventilator-associated pneumonia (VAP) and airway injury, which can negatively affect patient outcomes.^[4–6]^ In the ICU, the success rate of weaning from mechanical ventilation is directly related to patients’ quality of life and length of hospital stay.^[7–9]^ Improving the success rate of ventilator weaning and reducing complications associated with weaning failure have become major challenges in clinical research and practice.

In this context, targeted nursing, as a comprehensive and individualized intervention model, has gradually gained clinical attention. Unlike traditional nursing models, targeted nursing not only focuses on the patient’s physiological status but also emphasizes the patient’s psychological, social background, and disease characteristics, thereby developing personalized care plans.^[10]^ Several studies have demonstrated the significant advantages of this nursing model in improving clinical outcomes for ICU patients, particularly in the weaning process, where it can notably increase the success rate of ventilator weaning and reduce the risk of weaning failure.^[11]^ Despite existing evidence indicating that targeted nursing helps improve ventilator weaning success in ICU patients, its specific mechanisms and long-term effects on pulmonary function recovery have not been fully verified.

This study aims to explore the specific effects of targeted nursing on the ventilator weaning success rate and pulmonary function recovery in ICU patients with severe pneumonia. Through a retrospective cohort study, we observed the clinical outcomes of 2 groups of patients who received conventional nursing and targeted nursing interventions, including ventilator weaning success rates, pulmonary function recovery, and length of hospital stay. This study not only fills the gap in the current literature regarding the application of targeted nursing in severe pneumonia patients but also seeks to provide new insights into clinical nursing practice in the ICU. Ultimately, it aims to improve the success rate of ventilator weaning and the quality of life in severe pneumonia patients. Through this research, we hope to offer more targeted interventions in clinical care, facilitate early recovery, shorten hospital stays, and ultimately improve clinical prognosis.

## 2. Materials and methods

### 2.1. General information

This study is a retrospective cohort study involving 110 critically ill pneumonia patients who received mechanical ventilation in the ICU of our hospital from January 2022 to July 2024. Patients were divided into an observation group (n = 55) and a control group (n = 55) based on the nursing protocols. The observation group received targeted nursing interventions, combined with early rehabilitation training, while the control group received conventional nursing and standard early rehabilitation training. All data were collected through the hospital’s electronic health record system, ensuring the anonymity of the data to protect patient privacy and data security. Since this is a retrospective study, informed consent was archived in the hospital database, and no additional written consent was required from the patients.

### 2.2. Inclusion and exclusion criteria

#### 2.2.1. Inclusion criteria

Patients included in this study must meet the diagnostic criteria for severe pneumonia and exhibit varying degrees of mental lethargy, fatigue, hypotension, and respiratory failure. Patients with an oxygenation index ≤ 250 mm Hg, respiratory rate ≥ 30 breaths/min, and confirmed severe pneumonia via arterial blood gas analysis and imaging were included. All patients received invasive mechanical ventilation (i.e., mechanical ventilation via endotracheal intubation), and the mechanical ventilation duration was ≥24 hours. Additionally, the clinical data, including medical records, prescriptions, and test reports, were complete for all patients.

#### 2.2.2. Exclusion criteria

Exclusion criteria include patients with other severe respiratory diseases such as tuberculosis, or those with severe liver, kidney, or other organ dysfunction, as well as those with blood, immune, or endocrine system diseases. Patients with severe consciousness disorders, emotional disorders, or psychiatric histories who were unable to cooperate with basic research investigations were also excluded.

### 2.3. Nursing protocols

#### 2.3.1. Control group

The control group received conventional nursing care. During mechanical ventilation, the nursing team closely monitored the patient’s vital signs and clinical status, promptly informing the physician of any abnormalities. Ventilator settings were adjusted according to the patient’s condition, and ventilators were strictly disinfected. All patients received continuous care for a duration of 2 weeks.

#### 2.3.2. Observation group

The observation group received individualized nursing care, which was customized based on the patient’s specific needs, disease condition, and response to treatment. This approach differed significantly from the standard care provided to the control group and included the following detailed measures:

##### 2.3.2.1. Disease monitoring

Continuous, real-time assessment of vital signs using multifunctional monitors (e.g., body temperature, blood pressure, respiratory rate, and oxygen saturation) every 30 minutes. Adjustments to ventilator settings were made immediately based on changes in vital signs, blood gases, and clinical condition. Abnormalities such as fluctuations in blood pressure or reduced urine output triggered immediate communication with the physician for appropriate intervention. This level of frequent and tailored monitoring was not part of the standard care protocol.

##### 2.3.2.2. Airway, nasal, and oral care

Patients were provided with individualized airway and oral care to optimize respiratory function and minimize infections. Masks were selected based on the patient’s facial anatomy to reduce pressure sores, and respiratory secretions were cleared using a structured protocol. Oral care included the use of chlorhexidine mouthwash 3 times a day. Additionally, the frequency of oral care and airway management was adjusted according to the patient’s needs, which was in contrast to the routine care given in the control group.

##### 2.3.2.3. Respiratory function exercises

For patients who were able to breathe independently and were either extubated or preparing for extubation, specific respiratory rehabilitation exercises were implemented. These included structured abdominal breathing exercises to optimize lung expansion and oxygenation. Patients were instructed to place a hand on their abdomen, inhale deeply through the nose (allowing the abdomen to rise), and exhale slowly while pursing the lips, with a prescribed 3:1 inhale-exhale ratio. For intubated patients, passive breathing exercises were conducted, and ventilator settings were adjusted to encourage lung compliance. This active involvement in respiratory rehabilitation was a key component that distinguished individualized care from the passive approach in standard care.

##### 2.3.2.4. Pulmonary care

To prevent pulmonary complications, patients received tailored pulmonary care every 3 hours, including turning and percussion therapy (50–100 taps per minute on the lower lung lobes) to mobilize secretions and promote lung expansion. The specific timing, frequency, and technique of percussion therapy were adjusted according to each patient’s condition. Additionally, for patients with a persistent fever, alcohol wipes were used to manage body temperature. Regular bacterial cultures of tracheal secretions were taken, and antibiotics were administered based on the culture results and physician orders, rather than simply following standard antibiotic protocols.

##### 2.3.2.5. Weaning preparation

Prior to initiating ventilator weaning, specific preparations were made, including ensuring that simple resuscitators and respiratory stimulants were available. Detailed psychological support was provided to the patient by explaining the weaning process and ensuring a comfortable, relaxed position. Airway and oral secretions were cleared to facilitate the weaning process. Unlike standard care, where weaning is typically managed without a structured preparatory protocol, this individualized approach aimed to reduce anxiety and improve weaning success rates.

##### 2.3.2.6. Psychological care

Psychological support was tailored to each patient’s needs. Soothing music was played based on the patient’s preferences to help them relax and distract from the illness. Active conversations were encouraged for patients who could communicate freely, while nonverbal support such as hand-holding or gentle shoulder pats was provided to patients who had difficulty expressing themselves. This personalized psychological care was a core component of the individualized intervention, promoting both mental and emotional well-being.

All the above nursing measures were designed to be flexible and adapted based on the patient’s evolving condition. These interventions were provided for a duration of 2 weeks, aiming to improve clinical symptoms, accelerate recovery, and reduce complications, which contrasts with the more general care approach typically provided in the control group.

### 2.4. Evaluation metrics

The primary evaluation metrics of this study include clinical indicators, respiratory function, adverse events, ventilator weaning success rate, and nursing satisfaction, as described below:

#### 2.4.1. Clinical indicators

Clinical indicators include time to normalization of body temperature, mechanical ventilation time, and ICU length of stay. These indicators were used to assess the impact of different nursing interventions on the clinical recovery of patients.

#### 2.4.2. Respiratory function

Respiratory function was assessed through arterial blood gas analysis, primarily measuring PaO_2_, PaCO_2_, and SpO_2_. Arterial blood samples were collected before and after nursing interventions, and the results were analyzed using an automated blood gas analyzer to evaluate the impact of different nursing interventions on pulmonary function recovery, particularly oxygenation levels, CO_2_ removal, and blood oxygen saturation.

#### 2.4.3. Ventilator weaning success rate and complication incidence

The success rate of ventilator weaning was assessed by observing whether the patient could breathe smoothly, without signs of dyspnea or respiratory distress, within 48 hours postextubation, and whether there was hemodynamic stability and no abnormal arterial blood gas changes, with no need for reintubation. The incidence of complications, including (VAP), atelectasis, deep venous thrombosis (DVT), and delirium, was also recorded. Comparing the success rate of ventilator weaning and complication incidence between the 2 groups helps evaluate the effectiveness of nursing interventions in reducing complications and improving weaning success.

#### 2.4.4. Nursing satisfaction

Nursing satisfaction was assessed using a hospital nursing quality scoring table and nursing quality control evaluations by the head nurse. The scoring table covered aspects such as nursing details, nursing outcomes, service attitude, and scope of care, and was regularly evaluated by the head nurse. Given that some ICU patients might have consciousness disorders and be unable to complete satisfaction questionnaires on their own, family members were allowed to assist with completing the nursing satisfaction survey. All surveys and ratings were completed before patient discharge to ensure comprehensive and reliable data.

### 2.5. Data collection

Data collection was conducted through a combination of electronic medical records and questionnaire surveys. The research team extracted basic information such as age, gender, and primary disease type from the patients’ hospitalization records. For the primary outcome measures (time to normalization of body temperature, ICU length of stay, mechanical ventilation time, pulmonary function recovery, etc.), data were collected through clinical assessments, regular evaluations, and disease progress records. Nursing satisfaction was evaluated through self-designed questionnaires completed by patients at the end of the intervention, covering aspects such as nursing quality, communication, and intervention effects. All data were collected and recorded by research team members according to standardized procedures to ensure accuracy and consistency. Detailed training was provided to the research team to ensure the accuracy of data collection.

### 2.6. Statistical methods

Data analysis was performed using SPSS 22.0. Continuous data were expressed as mean ± SD and compared using independent t-tests for normally distributed data. For non-normally distributed data, Mann–Whitney *U* tests were used. Categorical data were analyzed with Chi-square tests (χ^2^). A significance level of *P* < .05 was used. *T*-tests compared continuous variables (e.g., ICU stay, ventilation time), and Chi-square tests compared categorical outcomes (e.g., ventilator weaning success). For multiple comparisons, Bonferroni correction was applied.

## 3. Results

### 3.1. Baseline data

This study included 110 ICU patients with severe pneumonia requiring endotracheal intubation, divided into 55 in the observation group and 55 in the control group. No significant differences were observed between the groups in terms of age, gender, height, weight, BMI, marital status, education level, or comorbidities (*P* > .05) (Table 1). These findings indicate comparability between the groups at baseline, ensuring a solid basis for further analysis.

**Table 1:** Baseline data (X ± SD, n/%).

	Observation group (n = 55)	Control group (n = 55)	*t*/χ^2^	*P*-value
Age (yr)	59.56 ± 3.24	59.62 ± 3.26	*t* = −0.097	.923
Gender			χ^2^ = 0.037	.847
Male	29 (52.73)	30 (54.55)		
Female	26 (47.27)	25 (45.45)		
Height (cm)	167.15 ± 7.47	167.30 ± 7.25	*t* = −0.107	.915
Weight (kg)	63.45 ± 10.52	64.39 ± 10.52	*t* = −0.468	.640
BMI (kg/m^2^)	22.54 ± 3.63	21.48 ± 4.37	*t* = 1.384	.169
Marital status			χ^2^ = 0.453	.912
Spinsterhood	6 (10.91)	8 (14.54)		
Married	36 (65.45)	33 (60.00)		
Divorced/widowed	13 (23.64)	14 (25.45)		
Education level			χ^2^ = 0.184	.912
Primary education	25 (45.45)	26 (47.27)		
Secondary education	24 (43.64)	22 (40.00)		
Higher education	6 (10.91)	7 (12.73)		
Comorbidities			χ^2^ = 0.150	.699
COPD	6 (10.91)	4 (7.27)		
Hypertension	25 (45.45)	26 (47.27)		
Diabetes	22 (40.00)	24 (43.64)		
Coronary heart disease	2 (3.64)	1 (1.82)		

BMI = body mass index, COPD = chronic obstructive pulmonary disease, SD = standard deviation.

### 3.2. Comparison of clinical indicators

**Figure 1.**
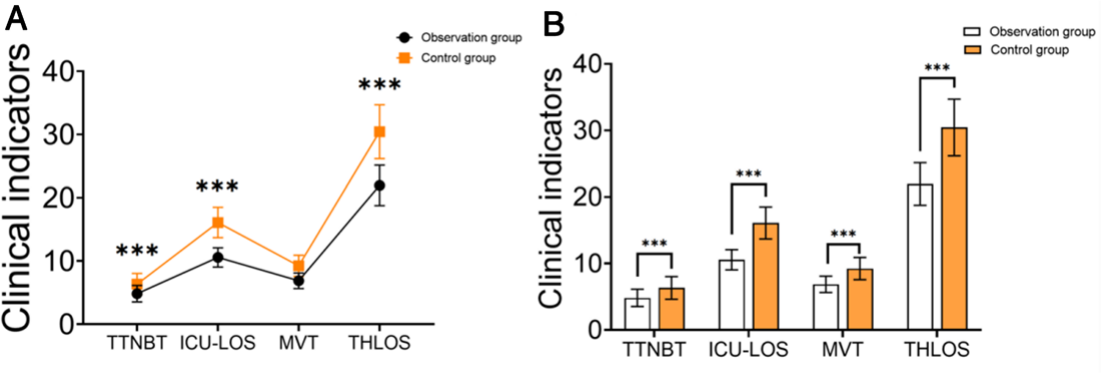
(A and B) Comparison of clinical indicators between the 2 groups.

The observation group had significantly shorter body temperature recovery time, ICU stay, mechanical ventilation duration, and total hospital stay compared to the control group. The observation group’s body temperature recovery time was 4.85 ± 1.30 hours, ICU stay was 10.58 ± 1.52 days, mechanical ventilation time was 6.88 ± 1.22 hours, and total hospital stay was 21.95 ± 3.20 days. In contrast, the control group’s corresponding times were 6.35 ± 1.70 hours, 16.10 ± 2.40 days, 9.25 ± 1.67 hours, and 30.45 ± 4.25 days. All differences were statistically significant (*P* < .05) (Table 2, Fig. 1). These results indicate that targeted nursing interventions can effectively shorten recovery time in severe pneumonia patients.

**Table 2:** Comparison of clinical indicators between the 2 groups (X ± SD).

Group	n	TTNBT	ICU-LOS	MVT	THLOS
Observation group	55	4.85 ± 1.30	10.58 ± 1.52	6.88 ± 1.22	21.95 ± 3.20
Control group	55	6.35 ± 1.70	16.10 ± 2.40	9.25 ± 1.67	30.45 ± 4.25
*T*		−5.190	−14.416	−8.496	−11.864
*P*		<.001	<.001	<.001	<.001

ICU-LOS = intensive care units-length of stay, MVT = mechanical ventilation time, SD = standard deviation, THLOS = total length of hospital stay, TTNBT = time to normalization of body temperature.

### 3.3. Comparison of respiratory function

**Figure 2.**
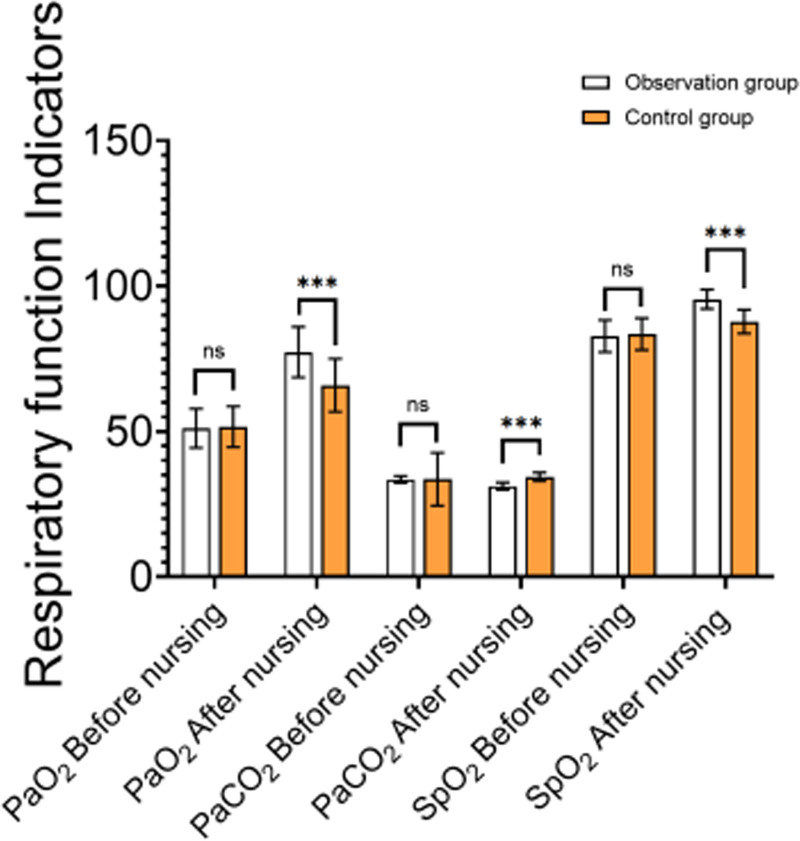
Comparison of respiratory function between the 2 groups.

Before the intervention, there were no significant differences between the observation and control groups in terms of PaO_2_, PaCO_2_, and SpO_2_ (*P* > .05). After the intervention, the observation group showed a significant improvement in PaO_2_ (77.30 ± 8.70 mm Hg vs 65.90 ± 9.10 mm Hg, *P* < .05). Moreover, PaCO_2_ decreased from 33.50 ± 1.20 mm Hg to 31.20 ± 1.30 mm Hg (*P* < .05), and SpO_2_ increased from 82.80 ± 5.50% to 95.50 ± 3.30% (*P* < .05) in the observation group. In comparison, the control group had a smaller decrease in PaCO_2_ (33.60 ± 1.90 mm Hg to 34.40 ± 1.50 mm Hg, *P* < .05) and a smaller increase in SpO_2_ (83.50 ± 4.90% to 87.80 ± 4.10%, *P* < .05) (Table 3, Fig. 2). These results indicate that targeted nursing interventions significantly improve respiratory function, particularly oxygenation and blood oxygen saturation, more effectively than conventional care.

**Table 3:** Comparison of respiratory function between the 2 groups (X ± SD, mm Hg).

Group	n	PaO_2_		PaCO_2_		SpO_2_	
		Before nursing	After nursing	Before nursing	After nursing	Before nursing	After nursing
Observation group	55	51.10 ± 6.70	77.30 ± 8.70	33.50 ± 1.20	31.20 ± 1.30	82.80 ± 5.50	95.50 ± 3.30
Control group	55	51.60 ± 7.00	65.90 ± 9.10	33.60 ± 1.90	34.40 ± 1.50	83.50 ± 4.90	87.80 ± 4.10
*t*		−0.382	6.710	−0.327	−7.110	−0.706	10.820
*P*		.703	<.001	.744	<.001	.481	<.001

SD = standard deviation.

### 3.4. Comparison of adverse events and ventilator weaning success rate

The observation group had a significantly lower overall adverse event rate of 10.91%, compared to 40.00% in the control group (*P* = .003). The incidence of deep venous thrombosis (DVT), bronchiectasis, (VAP), and ICU-acquired weakness (ICU-AW) in the observation group were 3.64%, 3.64%, 1.82%, and 1.82%, respectively, all lower than those in the control group (DVT 12.73%, bronchiectasis 7.27%, VAP 9.09%, ICU-AW 10.91%). Additionally, the observation group had a ventilator weaning success rate of 96.36%, significantly higher than the 81.82% in the control group (*P* = .014) (Table 4). These findings demonstrate that targeted nursing interventions significantly reduce the incidence of adverse events and improve the success rate of ventilator weaning.

**Table 4:** Comparison of adverse events and VRWS between 2 groups (n/%).

Group	n	Incidence of adverse events					VRWS
		DVT	Bronchiectasis	VAP	ICU-AW	Overall incidence rate	
Observation group	55	2 (3.64)	2 (3.64)	1 (1.82)	1 (1.82)	6 (10.91)	53 (96.36)
Control group	55	7 (12.73)	4 (7.27)	5 (9.09)	6 (10.91)	22 (40.00)	45 (81.82)
*t*		2.778	.666	2.666	3.572	9.142	6.000
*P*		.096	.415	.103	.059	.003	.014

DVT = deep venous thrombosis, ICU-AW = intensive care units-acquired weakness, VAP = ventilator-associated pneumonia, VRWS = success rate of ventilator weaning.

### 3.5. Comparison of nursing satisfaction

The observation group showed significantly higher overall nursing satisfaction (96.36%) compared to the control group (83.64%) (*P* = .026). In terms of “very satisfied” and “satisfied,” the observation group had 56.36% and 40.00%, respectively, while the control group had 50.91% and 32.73% (*P* > .05) (Table 5). These results suggest that targeted nursing interventions significantly enhance patient satisfaction.

**Table 5:** Comparison of nursing satisfaction between 2 groups (n/%).

Variables	Observation group (n = 55)	Control group (n = 55)	χ^2^	*P*
Dissatisfied	2 (3.64)	4 (7.27)	0.704	.401
Quite satisfied	22 (40.00)	18 (32.73)	0.628	.428
Very satisfied	31 (56.36)	28 (50.91)	0.329	.566
Overall satisfaction	53 (96.36)	46 (83.64)	4.948	.026

## 4. Discussion

Severe pneumonia is one of the leading causes of acute respiratory failure, often requiring mechanical ventilation to maintain vital signs.^[12,13]^ However, the success rate of weaning from mechanical ventilation and the occurrence of related complications remain significant factors affecting patient prognosis.^[14,15]^ The targeted nursing model, through individualized interventions, optimizes the overall care process, potentially accelerating patient recovery, reducing complications, and improving overall health and quality of life.

This retrospective cohort study evaluated the impact of targeted nursing on ventilator weaning success and pulmonary function recovery in patients with severe pneumonia. The results showed that the observation group significantly outperformed the control group in clinical indicators such as body temperature recovery time, ICU stay, mechanical ventilation duration, and total hospital stay, indicating that targeted nursing can accelerate patient recovery. Further analysis revealed that the ventilator weaning success rate in the observation group was 96.36%, significantly higher than the 81.82% in the control group. Additionally, the incidence of adverse events in the observation group was 10.91%, significantly lower than the 40.00% in the control group, suggesting that targeted nursing interventions effectively improve weaning success and reduce complications.

These results are consistent with existing literature, supporting the positive effects of individualized care on the recovery of critically ill patients. For example, several studies have shown that personalized care models, through multidisciplinary collaboration and optimized preoperative and postoperative management, can accelerate recovery and reduce the incidence of complications.^[16–18]^ Yu et al also found that the ERAS care model significantly improved recovery outcomes in various surgical procedures, including reducing postoperative complications and shortening hospital stays.^[19]^ This study validates the effectiveness of the targeted nursing model in severe pneumonia patients, particularly in improving ventilator weaning success and controlling complications.

Additionally, this study provides a detailed evaluation of the impact of this nursing model on patient psychological status and nursing satisfaction. The results showed that the observation group had significantly higher nursing satisfaction than the control group, indicating that targeted nursing interventions effectively enhance patients’ subjective experiences and satisfaction with nursing services. This finding aligns with previous studies, suggesting that personalized care improves overall patient satisfaction and increases their recognition of the care provided.^[20]^

Despite the positive outcomes, there are some limitations to this study. First, the retrospective cohort design may introduce selection and information biases, potentially affecting the reliability of the results. Secondly, the study was conducted at a single center, which limits the generalizability of the findings. Future multi-center, prospective studies are needed to further verify the effectiveness and broader applicability of this nursing model, with the goal of benefiting more patients in wider clinical settings.

## 5. Conclusion

This study confirms that targeted nursing offers significant advantages in ventilator weaning, pulmonary function recovery, complication control, and nursing satisfaction in patients with severe pneumonia. This nursing model not only accelerates patient recovery, reduces complications, and enhances quality of life but also improves patient satisfaction with nursing care. Although this study has some limitations, its findings provide strong evidence for clinical practice. Future large-scale, prospective studies are needed to further validate the effectiveness and broader applicability of this nursing model, with the aim of benefiting more patients in a wider clinical context.

## Author contributions

**Conceptualization:** Liangyou Zheng, Ni Li.

**Data curation:** Ni Li, Li Zhao, Sha Hu.

**Investigation:** Ni Li, Xiaofeng Zhong.

**Methodology:** Liangyou Zheng, Chunlan Zheng.

**Software:** Liangyou Zheng, Wen Li.

**Supervision:** Liangyou Zheng, Bei Pan.

**Writing – original draft:** Ni Li.

**Writing – review & editing:** Liangyou Zheng.

## References

[1] Valade S Biard L Lemiale V. Severe atypical pneumonia in critically ill patients: a retrospective multicenter study. Ann Intensive Care. 2018;8:81. 30105627 10.1186/s13613-018-0429-z PMC6089852

[2] Miller PE Van Diepen S Metkus TS. Association between respiratory failure and clinical outcomes in patients with acute heart failure: analysis of 5 pooled clinical trials. J Card Fail. 2021;27:602–6. 33556546 10.1016/j.cardfail.2021.01.018 PMC8527461

[3] Sardu C D’Onofrio N Balestrieri ML. Outcomes in patients with hyperglycemia affected by COVID-19: can we do more on glycemic control? Diabetes Care. 2020;43:1408–15. 32430456 10.2337/dc20-0723 PMC7305003

[4] Heo SM Haase EM Lesse AJ Gill SR Scannapieco FA. Genetic relationships between respiratory pathogens isolated from dental plaque and bronchoalveolar lavage fluid from patients in the intensive care unit undergoing mechanical ventilation. Clin Infect Dis. 2008;47:1562–70. 18991508 10.1086/593193 PMC3582026

[5] Binnekade JM Tepaske R Bruynzeel P Mathus-Vliegen EM de Hann RJ. Daily enteral feeding practice on the ICU: attainment of goals and interfering factors. Crit Care. 2005;9:R218–25. 15987393 10.1186/cc3504 PMC1175883

[6] Miller PE van Diepen S Ahmad T. Acute decompensated heart failure complicated by respiratory failure. Circ Heart Fail. 2019;12:e006013. 31030542 10.1161/CIRCHEARTFAILURE.119.006013

[7] Hamon A Scemama U Bourenne J. Chest CT scan and alveolar procollagen III to predict lung fibroproliferation in acute respiratory distress syndrome. Ann Intensive Care. 2019;9:42. 30919111 10.1186/s13613-019-0516-9 PMC6437222

[8] Werho DK Fisk A Yeh J. Measuring critical care unit performance using a postoperative mechanical ventilation quality metric. Ann Thorac Surg. 2024;117:440–7. 36470563 10.1016/j.athoracsur.2022.11.026

[9] Herridge MS Tansey CM Matté A; Canadian Critical Care Trials Group. Functional disability 5 years after acute respiratory distress syndrome. N Engl J Med. 2011;364:1293–304. 21470008 10.1056/NEJMoa1011802

[10] Simpson HB Maher MJ Wang Y Bao Y Foa EB Franklin M. Patient adherence predicts outcome from cognitive behavioral therapy in obsessive-compulsive disorder. J Consult Clin Psychol. 2011;79:247–52. 21355639 10.1037/a0022659 PMC3891521

[11] Bento AFG Sousa PP. Delirium in adult patients in intensive care: nursing interventions. Br J Nurs. 2021;30:534–8. 33983821 10.12968/bjon.2021.30.9.534

[12] Dai YJ Zhang WN Wang WD He SY Liang CC Wang DW. Comprehensive analysis of two potential novel SARS-CoV-2 entries, TMPRSS2 and IFITM3, in healthy individuals and cancer patients. Int J Biol Sci. 2020;16:3028–36. 33061814 10.7150/ijbs.51234 PMC7545701

[13] Luo J Yu H Hu YH. Early identification of patients at risk for acute respiratory distress syndrome among severe pneumonia: a retrospective cohort study. J Thorac Dis. 2017;9:3979–95. 29268409 10.21037/jtd.2017.09.20 PMC5723858

[14] Yeung J Couper K Ryan EG Gates S Hart N Perkins GD. Non-invasive ventilation as a strategy for weaning from invasive mechanical ventilation: a systematic review and Bayesian meta-analysis. Intensive Care Med. 2018;44:2192–204. 30382306 10.1007/s00134-018-5434-z PMC6280833

[15] Nakanishi N Oto J Ueno Y Nakataki E Itagaki T Nishimura M. Change in diaphragm and intercostal muscle thickness in mechanically ventilated patients: a prospective observational ultrasonography study. J Intensive Care. 2019;7:56. 31827804 10.1186/s40560-019-0410-4 PMC6886193

[16] Ji HB Zhu WT Wei Q Wang XX Wang HB Chen QP. Impact of enhanced recovery after surgery programs on pancreatic surgery: a meta-analysis. World J Gastroenterol. 2018;24:1666–78. 29686474 10.3748/wjg.v24.i15.1666 PMC5910550

[17] Bateni SB Olson JL Hoch JS Canter RJ Bold RJ. Drivers of cost for pancreatic surgery: it’s not about hospital volume. Ann Surg Oncol. 2018;25:3804–11. 30218244 10.1245/s10434-018-6758-1 PMC6367004

[18] Lloyd CE Pambianco G Orchard TJ. Does diabetes-related distress explain the presence of depressive symptoms and/or poor self-care in individuals with type 1 diabetes? Diabet Med. 2010;27:234–7. 20546270 10.1111/j.1464-5491.2009.02896.x PMC3093054

[19] Yu P Wang G Zhang C. Clinical application of enhanced recovery after surgery (ERAS) in pectus excavatum patients following Nuss procedure. J Thorac Dis. 2020;12:3035–42. 32642226 10.21037/jtd-20-1516 PMC7330763

[20] Mukamba N Chilyabanyama ON Beres LK. Patients’ satisfaction with HIV care providers in public health facilities in lusaka: a study of patients who were lost-to-follow-up from HIV care and treatment. AIDS Behav. 2020;24:1151–60. 31673912 10.1007/s10461-019-02712-4 PMC7082366

